# PRPF19 regulates p53-dependent cellular senescence by modulating alternative splicing of *MDM4* mRNA

**DOI:** 10.1016/j.jbc.2021.100882

**Published:** 2021-06-16

**Authors:** Kimiyoshi Yano, Ryou-u Takahashi, Bunsyo Shiotani, Junko Abe, Tomoki Shidooka, Yuki Sudo, Yusuke Yamamoto, Shisei Kan, Hiroki Sakagami, Hidetoshi Tahara

**Affiliations:** 1 Department of Cellular and Molecular Biology, Graduate School of Biomedical Sciences, Hiroshima University, Hiroshima, Japan; 2 Division of Cellular Signaling, National Cancer Center Research Institute, Tokyo, Japan

**Keywords:** fibroblast, cellular senescence, RNA processing, RNA splicing, DNA damage response, p53, alternative splicing, DDR, DNA damage response, MATS, multivariate analysis of transcript splicing, MDM, murine double minute, PDL, population doubling level, SAHF, senescence-associated heterochromatin focus, SRSF3, serine/arginine-rich splicing factor 3

## Abstract

Alteration of RNA splicing is a hallmark of cellular senescence, which is associated with age-related disease and cancer development. However, the roles of splicing factors in cellular senescence are not fully understood. In this study, we identified the splicing factor PRPF19 as a critical regulator of cellular senescence in normal human diploid fibroblasts. PRPF19 was downregulated during replicative senescence, and PRPF19 knockdown prematurely induced senescence-like cell cycle arrest through the p53–p21 pathway. RNA-sequencing analysis revealed that PRPF19 knockdown caused a switch of the *MDM4* splicing isoform from stable full-length MDM4-FL to unstable MDM4-S lacking exon 6. We also found that PRPF19 regulates *MDM4* splicing by promoting the physical interaction of other splicing factors, PRPF3 and PRPF8, which are key components of the core spliceosome, U4/U6.U5 tri-snRNP. Given that MDM4 is a major negative regulator of p53, our findings imply that PRPF19 downregulation inhibits MDM4-mediated p53 inactivation, resulting in induction of cellular senescence. Thus, PRPF19 plays an important role in the induction of p53-dependent cellular senescence.

Cellular senescence is a prolonged state of proliferative arrest caused by various types of stress, including telomere shortening, DNA damage, or oncogenic stress (1). The cell cycle arrest in senescent cells is mediated mainly by tumor suppressors such as p53, p21, and p16 (2). In addition to cell cycle arrest, cellular senescence is characterized by morphological changes, high β-galactosidase activity, and chromatin remodeling (3, 4, 5). Senescent cells with these phenotypic alterations are observed in the tumor environment, embryonic development, and tissue repair (6), suggesting that cellular senescence plays important roles in multiple aspects of cellular and tissue homeostasis. Elucidation of the underlying molecular mechanisms would contribute to our understanding of the biological functions of cellular senescence in normal development and disease progression.

Since p53 is negatively regulated by murine double minute 2 (MDM2) and murine double minute 4 (MDM4, also known as MDMX) (7), inhibition of MDM2 and MDM4 results in p53-mediated antiproliferative activity, including apoptosis and cellular senescence (8, 9). MDM2 has E3 ubiquitin ligase activity and degrades p53 *via* the proteasome (10), whereas MDM4, which has no E3 activity, promotes MDM2-mediated degradation of p53 and inhibits p53 transcriptional activity (11). The *MDM4* gene produces two main splicing isoforms: a stable transcript including the full-length protein-coding sequence (MDM4-FL) and an unstable transcript lacking exon 6 (MDM4-S). In melanoma, alternative splicing of *MDM4* mRNA is regulated by serine/arginine-rich splicing factor 3 (SRSF3), which belongs to the SR protein family of splicing factors (12). SRSF3 depletion promotes generation of the MDM4-S isoform and inhibits tumor cell proliferation by inducing apoptosis. Since MDM4 is more highly expressed in cancer tissues than in normal tissues and dampens the tumor-suppressive functions of p53 (13, 14, 15, 16), alternative splicing of *MDM4* mRNA is considered to be a critical step in cancer progression (17). However, it remains unknown whether alternative splicing of *MDM4* mRNA contributes to cellular senescence.

The DNA damage response (DDR) also regulates cellular senescence through activation of the p53–p21 pathway (18, 19, 20). The DDR is mediated by ataxia telangiectasia–mutated (ATM) and ataxia telangiectasia and Rad3-related (ATR) proteins, which belong to the phosphatidylinositol 3-kinase family (21). ATM and ATR phosphorylate downstream effectors such as checkpoint kinase 1/2 (Chk1/2), leading to p53 activation (22). Moreover, the DDR alters the expression of splicing factors and the alternative splicing of mRNAs involved in the cell cycle and apoptosis (23). The DDR promotes generation of the p53β isoform, an alternative splicing product of the *TP53* gene, resulting in cellular senescence (24). Although several studies have reported that alteration of splicing factors and alternative splicing isoforms is important for cellular senescence (25), their mechanisms are not yet fully understood.

In this study, we investigated the roles of splicing factors in cellular senescence of normal human diploid fibroblasts. Our microarray analysis identified pre-mRNA processing factor 19 (PRPF19) as a key splicing factor in cellular senescence. PRPF19 expression was reduced at the mRNA and protein levels during replicative senescence. In addition, PRPF19 knockdown caused a switch of the *MDM4* splicing isoform from MDM4-FL to MDM4-S, resulting in p53-dependent cellular senescence. We also found that PRPF19 indirectly modulated the *MDM4* alternative splicing by promoting the physical interaction of other splicing factors, PRPF3 and PRPF8, which are key components of the core spliceosome, U4/U6.U5 tri-snRNP (26). Thus, our findings provide new insights into the roles of splicing factors in the regulation of cellular senescence.

## Results

### Functional screening for senescence-associated splicing factors

**Figure 1:**
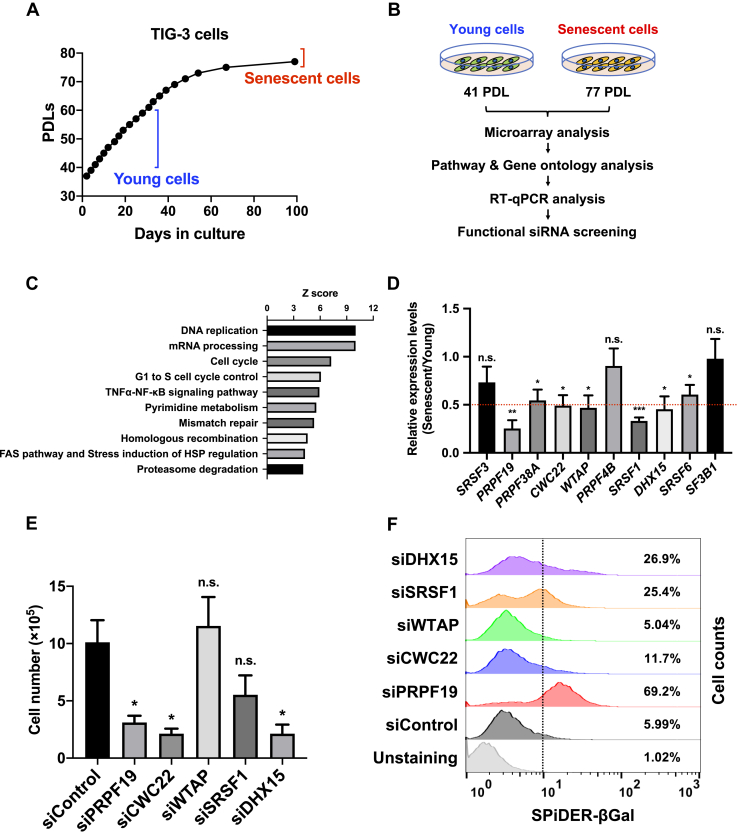
**Identification of PRPF19 as a key regulator of cellular senescence.*****A*****,** population doubling levels (PDLs) of TIG-3 cells used in microarray experiments. ***B*,** experimental design for identification of novel senescence regulators. Differences in gene expression between young (PDL 41) and senescent (PDL 77) cells were analyzed by microarray. ***C*,** top ten significant pathways containing genes whose expression was downregulated by more than 50% in senescent cells. ***D*,** relative quantification by RT-qPCR of the top ten genes involved in RNA splicing and processing that were downregulated in senescent cells, as determined by microarray. Expression levels of each gene were normalized against the corresponding level of *GAPDH*. Red bar indicates 0.5-fold. All values represent means ± SD of three independent experiments. ∗*p* < 0.05, ∗∗*p* < 0.01, n.s. (not significant); two-tailed Student’s *t* test. ***E*,** bar graph indicates relative cell viability in TIG-3 cells 4 days after transfection with the indicated siRNAs. All values represent means ± SD of three independent experiments. ∗*p* < 0.05, n.s. (not significant); two-tailed Student’s *t* test. ***F*,** histogram shows fluorescence-based SA-β-gal activity of TIG-3 cells 7 days after transfection with the indicated siRNAs.

To identify splicing factors associated with cellular senescence, we first performed microarray analysis to compare early-passage (population doubling level [PDL] = 41) and late-passage (PDL = 77) TIG-3 normal human diploid fibroblasts (Fig. 1, *A* and *B*). Consistent with a previous study (27), pathway and Gene Ontology analysis of differentially expressed genes revealed downregulation of gene sets involved in mRNA processing and splicing, as well as DNA replication and the cell cycle, during replicative senescence (Fig. 1*C* and S1A). To validate the changes in expression detected by microarray, we performed reverse transcription–quantitative PCR (RT-qPCR). The RT-qPCR analysis confirmed that seven genes (*PRPF19*, *PRPF38A*, *CWC22*, *WTAP*, *SRSF1*, *DHX15*, and *SRSF6*) were significantly downregulated in senescent TIG-3 cells compared with young TIG-3 cells (Fig. 1*D*). Among the downregulated genes, we focused on five (*PRPF19*, *CWC22*, *WTAP*, *SRSF1*, and *DHX15*) whose expression was reduced by more than 50% in senescent cells (Fig. 1*D* and S1B).

We next performed functional siRNA screening to determine whether depletion of candidate genes contributes to the induction of cellular senescence in TIG-3 cells. For these experiments, we first prepared three siRNAs against each candidate gene and evaluated their knockdown efficacy (Fig. S1, *C* and *D*). Using the siRNAs with the highest knockdown efficacy, we examined senescent phenotypes such as cell proliferation and senescence-associated β-galactosidase (SA-β-gal) activity. Knockdown of PRPF19, CWC22, and DHX15 significantly inhibited the proliferation of TIG-3 cells (Fig. 1*E*). Importantly, flow cytometry analysis revealed that PRPF19 knockdown most strongly increased the proportion of TIG-3 cells with high SA-β-gal activity among these five candidate genes (Fig. 1*F*). These results suggest that PRPF19 plays an important role in the induction of cellular senescence.

### PRPF19 is a critical regulator of cellular senescence

**Figure 2:**
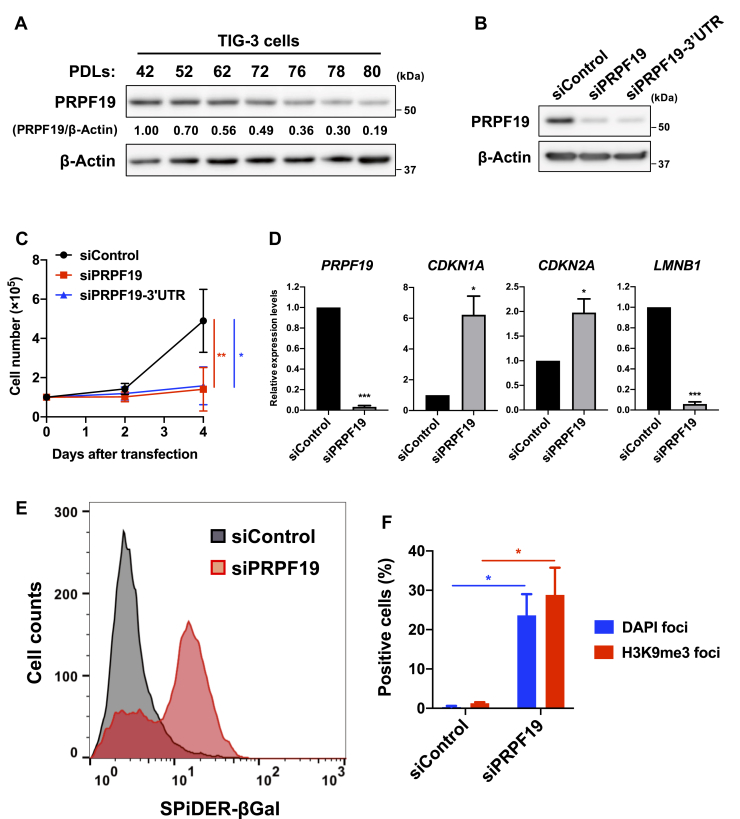
**Depletion of PRPF19 induces cellular senescence in human fibroblasts. *A*,** immunoblot analysis of TIG-3 cells at the indicated PDLs. Relative levels of PRPF19 were normalized against the corresponding levels of β-Actin. Intensity was calculated using NIH ImageJ. ***B*,** immunoblot analysis of TIG-3 cells 3 days after transfection with the indicated siRNAs. ***C*,** cell growth curve in TIG-3 cells. Cells were counted at the indicated time points after transfection with the indicated siRNAs. All values represent means ± SD of three independent experiments. ∗*p* < 0.05, ∗∗*p* < 0.01; two-tailed Student’s *t* test. ***D*,** relative quantification by RT-qPCR of TIG-3 cells 7 days after transfection with the indicated siRNAs. Expression levels of each gene were normalized against the corresponding level of *GAPDH*. Data represent the means ± SD of three independent experiments. ∗*p* < 0.05, ∗∗∗*p* < 0.001; two-tailed Student’s *t* test. ***E*,** histogram shows fluorescence-based SA-β-gal activity of TIG-3 cells 7 days after transfection with the indicated siRNAs. ***F*,** bar graph shows percentage of TIG-3 cells positive for DAPI and H3K9me3 foci 7 days after transfection with the indicated siRNAs. All values represent means ± SD of three independent experiments. ∗*p* < 0.05; two-tailed Student’s *t* test.

To further investigate whether PRPF19 depletion induces cellular senescence, we evaluated several markers of cellular senescence using TIG-3 cells, in which PRPF19 was downregulated during cellular senescence (Fig. 2*A*). For these experiments, we used two types of siRNAs against *PRPF19*: one targeting the coding region (siPRPF19) and the other targeting its 3’-untranslated region (siPRPF19–3’UTR). Both siRNAs efficiently downregulated the level of PRPF19 protein and significantly suppressed cell proliferation in young TIG-3 cells (Fig. 2, *B* and *C*). In addition, we examined the changes in expression of senescence-associated genes such as *CDKN1A* (encoding cyclin-dependent kinase inhibitor p21), *CDKN2A* (encoding cyclin-dependent kinase inhibitor p16), and *LMNB1* (encoding nuclear lamina component lamin B1), because upregulated levels of *CDKN1A* and *CDKN2A* and downregulated levels of *LMNB1* are typical makers of cellular senescence (28, 29). At 7 days after transfection with the siRNAs, RT-qPCR analysis revealed upregulation of *CDKN1A* and *CDKN2A* and downregulation of *LMNB1* after PRPF19 knockdown (Fig. 2*D*). We also found that PRPF19 knockdown increased the proportion of TIG-3 cells with high SA-β-gal activity and trimethylation of histone H3 Lys 9 (H3K9me3), both of which are well-characterized markers of cellular senescence (Fig. 2, *E* and *F*, and Fig. S2A). In addition, we observed that PRPF19 knockdown suppressed cell proliferation and increased SA-β-gal activity in other normal human diploid fibroblasts, MRC-5 and IMR-90, as well as in TIG-3 cells (Fig. S2, *B–E*). Considering the significant changes in multiple markers of cellular senescence after PRPF19 knockdown, these results indicate that PRPF19 downregulation induces cellular senescence in human fibroblasts.

### PRPF19 downregulation triggers p53-dependent cellular senescence

**Figure 3:**
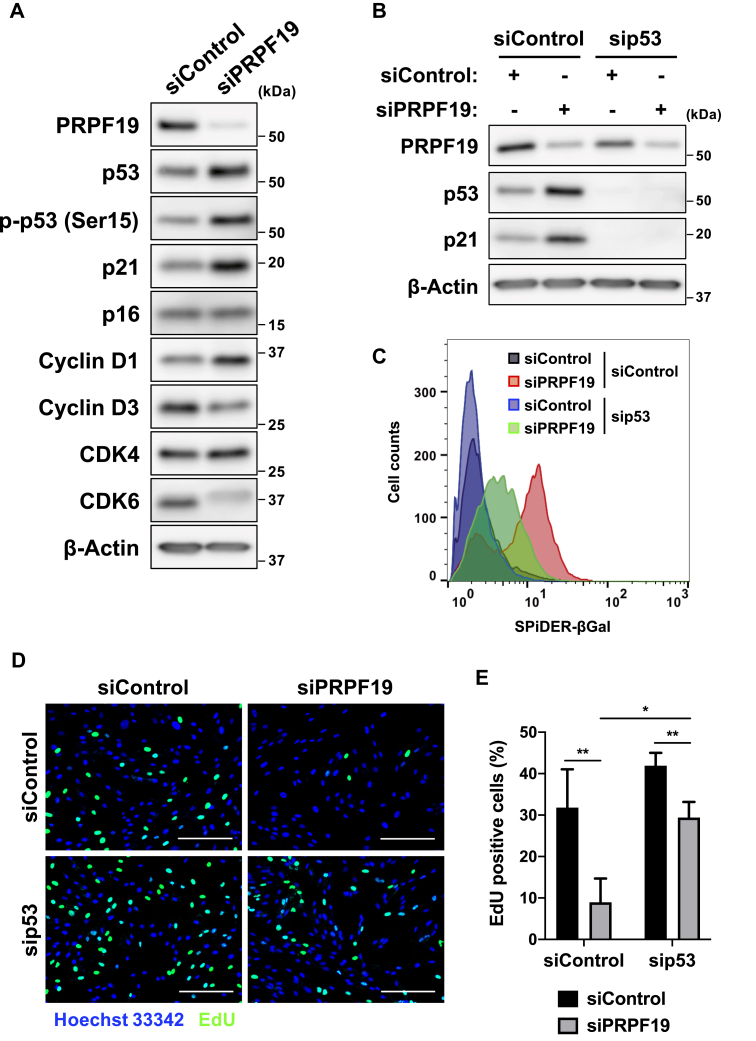
**p53 is required for senescence-like cell cycle arrest in PRPF19-depleted cells.*****A*****,** immunoblot analysis of TIG-3 cells 3 days after transfection with the indicated siRNAs. ***B*,** immunoblot analysis of TIG-3 cells 2 days after transfection with siControl or siPRPF19. Cells were pretransfected with siControl or sip53 2 days prior to transfection with siControl or siPRPF19. ***C*,** histogram shows fluorescence-based SA-β-gal activity of TIG-3 7 days after transfection with siControl or siPRPF19. Cells were pretransfected with siControl or sip53 2 days prior to transfection with siControl or siPRPF19. ***D*,** fluorescence microphotographs of TIG-3 cells in EdU cell proliferation assay. Cells were pretransfected with siControl or sip53 2 days prior to transfection with siControl or siPRPF19. Two days after transfection with siControl or siPRPF19, cells were cultured in growth medium supplemented with 10 μM EdU for 24 h. Scale bar, 200 μm. ***E*,** bar graph shows percentage of EdU-positive cells in the experiment shown in Figure 3*D*. All values represent means ± SD of three independent experiments. ∗*p* < 0.05, ∗∗*p* < 0.01; two-tailed Student’s *t* test.

To understand the molecular mechanisms by which PRPF19 downregulation induces cellular senescence, we examined the changes in expression of several G1/S cell cycle regulators after PRPF19 knockdown. Three days after transfection, immunoblot and RT-qPCR analysis revealed that PRPF19 knockdown stimulated activation of the p53–p21 pathway, as indicated by stabilized and phosphorylated p53 and upregulated p21 (Fig. 3*A*). In comparison, PRPF19 knockdown had little effect on expression of p16, CDK4, and cyclin D1 for at least 3 days after transfection (Fig. 3*A*). Therefore, we hypothesized that PRPF19 downregulation induces cellular senescence through activation of the p53–p21 pathway.

To test this hypothesis, we examined the effects of PRPF19 knockdown in p53-depleted TIG-3 cells. p53 depletion abolished the upregulation of p21 by PRPF19 knockdown (Fig. 3*B*). To determine whether p53 is associated with PRPF19-mediated cellular senescence, we performed SA-β-gal assays after PRPF19 knockdown in p53-depleted cells. Flow cytometry revealed low SA-β-gal activity after PRPF19 knockdown in p53-depleted cells compared with control cells (Fig. 3*C*). In addition, we performed a cell proliferation assay using 5-ethynyl-2’-deoxyuridine (EdU), a thymidine analog that is incorporated into DNA during S phase. We observed that p53 depletion significantly restored the decrease in the proportion of EdU-positive cells caused by PRPF19 knockdown (Fig. 3, *D* and *E*), suggesting that p53 is required for cell cycle arrest in PRPF19-depleted cells. Taken together, these observations indicate that PRPF19 downregulation induces p53-dependent cellular senescence.

### The DDR is not involved in PRPF19-mediated cellular senescence

**Figure 4:**
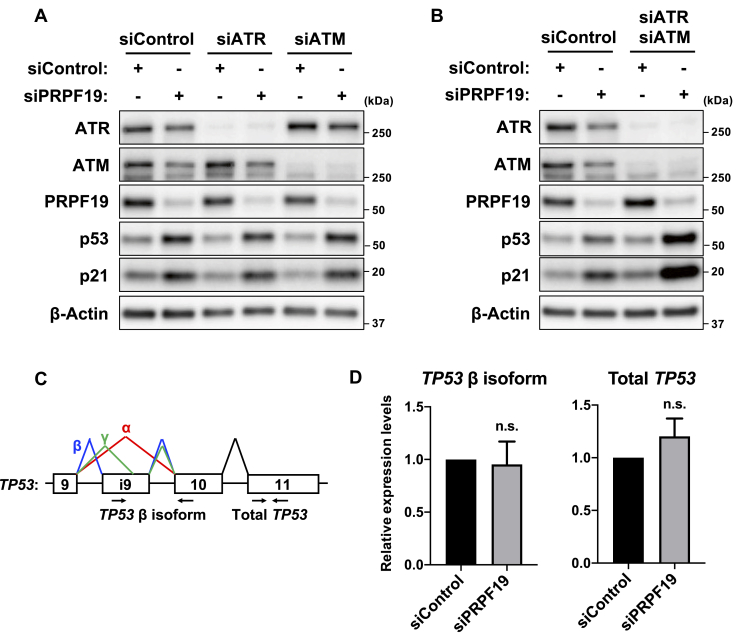
**Depletion of PRPF19 stimulates activation of the p53–p21 pathway independently of ATR and ATM**. ***A*** and ***B*,** immunoblot analysis of TIG-3 cells 3 days after cotransfection with the indicated siRNAs. ***C*,** expression pattern of *TP53* mRNA. PCR primers were designed to detect total *TP53* mRNA and the *TP53*β isoform. ***D*,** expression levels of *TP53* mRNA and the *TP53*β isoform. RT-qPCR analysis of TIG-3 cells 3 days after transfection with the indicated siRNAs. Expression levels of each gene were normalized against the corresponding level of *GAPDH*. Data represent the means ± SD of three independent experiments. n.s. (not significant); two-tailed Student’s *t* test.

Since DNA damage is often observed in senescent cells, in which the DDR stimulates activation of the p53–p21 pathway (18), we investigated the relationship between the DDR and PRPF19 in cellular senescence. During DNA end resection, a broken DNA end is converted into a long stretch of single-stranded DNA with a 3’-overhang, which is detectable by 5-bromo-2’-deoxyuridine (BrdU) staining (30). Therefore, we performed immunostaining with anti-BrdU antibody after PRPF19 knockdown in TIG-3 cells. Flow cytometry revealed an increase in BrdU fluorescence intensity after PRPF19 knockdown (Fig. S3*A*). We also performed comet assays and quantified the percentage of DNA in comet tails to measure the levels of DNA damage in individual cells (31). The results revealed that PRPF19 knockdown significantly induced the accumulation of DNA damage (Fig. S3, *B–D*). ATM and ATR are primarily responsible for phosphorylation of p53 at Ser 15 in response to DNA damage (32, 33, 34). Since PRPF19 knockdown induced phosphorylation of p53 at Ser15 (Fig. 3*A*), we investigated whether ATM or ATR mediated activation of the p53–p21 pathway in PRPF19-depleted cells. Unexpectedly, immunoblot analysis revealed that knockdown of ATM and/or ATR had little effect on activation of the p53–p21 pathway by PRPF19 knockdown (Fig. 4, *A* and *B*).

Moreover, other studies reported that the p53β isoform induces cellular senescence as a component of the DDR (24). Therefore, we also examined the changes in expression of the *TP53* splicing isoforms by RT-PCR and qPCR analysis (Figs. 4*C* and S3*E*). Consistent with a previous study (35), SRSF3 knockdown dramatically promoted generation of the *TP53* β isoform (Fig. S3, *F* and *G*). Considering the knockdown efficacy of SRSF3, we confirmed an inverse correlation between the *TP53* β isoform and SRSF3 expression (Fig. S3*H*). By contrast, PRPF19 knockdown had little effect on expression of the *TP53* β isoform, as well as total *TP53* mRNA (Fig. 3*D* and S3, *F*, *G*, and *I*). Taken together, these results suggest that PRPF19 regulates p53-dependent cellular senescence independently of the DDR factors, including ATM, ATR, and p53β.

### PRPF19 downregulation stimulates activation of the p53–p21 pathway by promoting generation of the MDM4-S isoform

**Figure 5:**
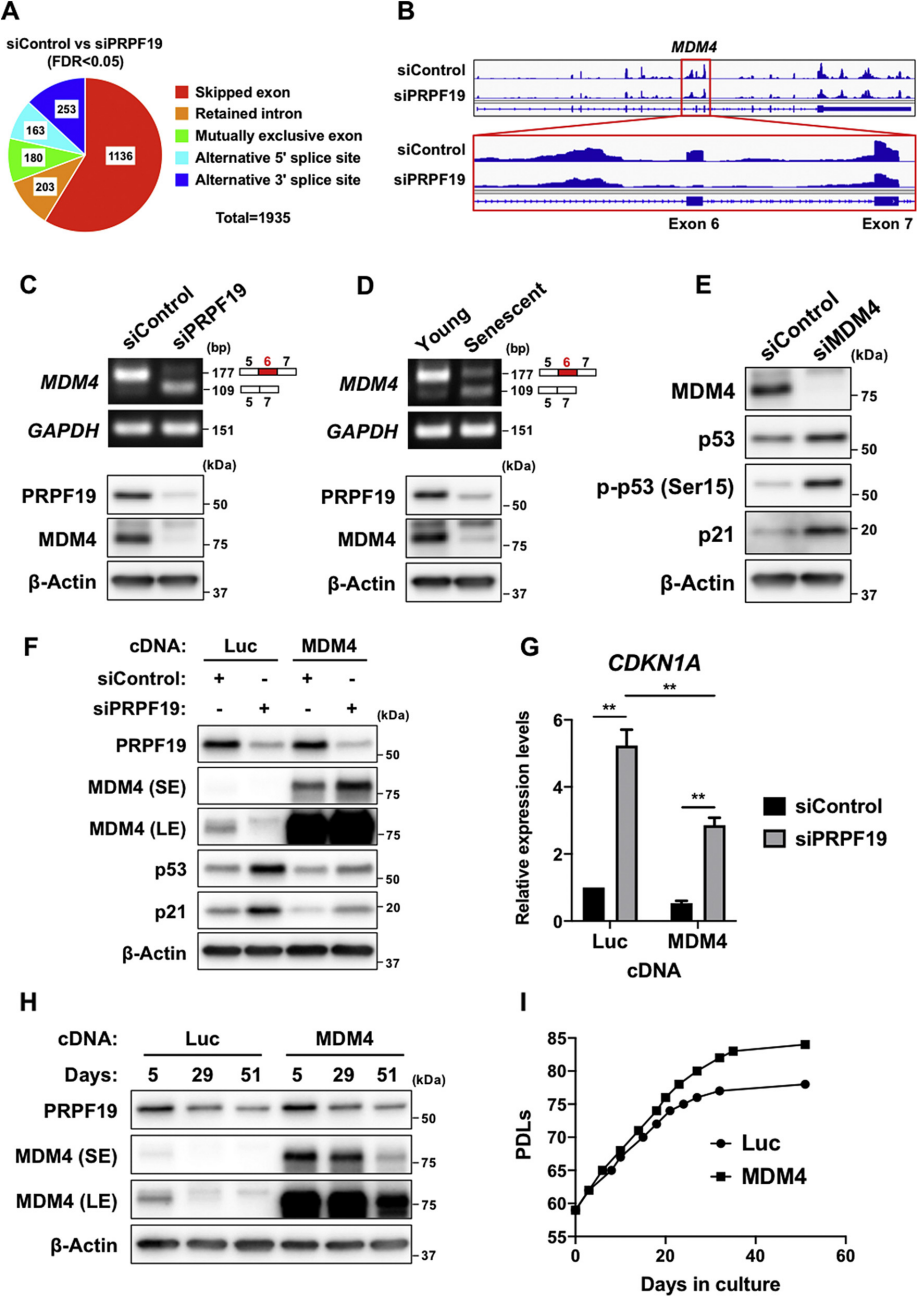
**PRPF19 is necessary for inclusion of exon 6 in the *MDM4* mRNA and full expression of MDM4 protein**. ***A*,** pie chart shows five types of alternative splicing events identified by RNA-seq analysis of TIG-3 cells 3 days after transfection with the indicated siRNAs. ***B*,** genome annotations acquired from RNA-seq analysis representing *MDM4* alternative splicing. *Red box* highlights the location of exon 6 skipping in the *MDM4* mRNA. ***C*,** RT-PCR analysis (*top*) and immunoblot analysis (*bottom*) of TIG-3 cells 3 days after transfection with the indicated siRNAs. ***D*,** RT-PCR analysis (*top*) and immunoblot analysis (*bottom*) of young and senescent TIG-3 cells. ***E*,** immunoblot analysis of TIG-3 cells 3 days after transfection with the indicated siRNAs. ***F*,** immunoblot analysis of luciferase- or MDM4-expressing TIG-3 cells 3 days after transfection with the indicated siRNAs. ***G*,** relative quantification by RT-qPCR analysis of luciferase- or MDM4-expressing TIG-3 cells 3 days after transfection with the indicated siRNAs. Expression levels of *CDKN1A* were normalized against the corresponding levels of *GAPDH*. All values represent means ± SD of three independent experiments. ∗∗*p* < 0.01; two-tailed Student’s *t* test. ***H*,** immunoblot analysis of luciferase- or MDM4-expressing TIG-3 cells at the indicated time points. ***I*,** luciferase- or MDM4-expressing TIG-3 cell cultures. LE, long exposure; SE, short exposure.

Since PRPF19 plays important roles in pre-mRNA splicing (36, 37), we next explored the possibility that PRPF19 downregulation affects the alternative splicing of senescence-associated genes. To identify splicing events altered by PRPF19 downregulation, we performed multivariate analysis of transcript splicing (MATS) using RNA-sequencing (RNA-seq) analysis. The result revealed changes in the splicing patterns of 1935 events after PRPF19 knockdown, consisting mainly of skipped exons (Fig. 5*A*). Among the candidate genes, we focused on *MDM4*, in which exon 6 was skipped after PRPF19 knockdown (Fig. 5*B*). MDM4, like MDM2, is a major negative regulator of p53 (7). In melanoma, SRSF3 depletion causes a switch of the *MDM4* splicing isoform from stable full-length MDM4-FL to unstable MDM4-S lacking exon 6, leading to p53-mediated antiproliferative activity (12). Hence, these findings prompted us to examine the function of *MDM4* alternative splicing in PRPF19-depleted and senescent TIG-3 cells.

To validate the altered splicing of the *MDM4* mRNA in PRPF19-depleted cells, we performed RT-PCR analysis (Fig. S4*A*). Consistent with the RNA-seq analysis, RT-PCR analysis revealed that PRPF19 knockdown caused exon 6 skipping in the *MDM4* mRNA, as indicated by downregulation of the MDM4-FL isoform and upregulation of the MDM4-S isoform (Fig. 5*C*, upper panel). In addition, immunoblot analysis revealed a significant reduction in the level of MDM4 protein after PRPF19 knockdown (Fig. 5*C*, lower panel). We obtained similar results in senescent TIG-3 cells (Fig. 5*D*). Consistent with these observations, MDM4 knockdown stimulated activation of the p53–p21 pathway (Fig. 5, *E*). Importantly, we obtained similar results in MRC-5 and IMR-90 cells (Fig. S4, *B* and *C*).

To further confirm the effect of PRPF19 on *MDM4* alternative splicing, we performed a rescue experiment in which the siRNAs were transfected into luciferase-expressing or 3’UTR-lacking PRPF19-expressing TIG-3 cells. Exogenous expression of siRNA-resistant PRPF19 inhibited the switch of the MDM4 splicing isoform after transfection with siPRPF19-3’UTR (Fig. S4*D*). These observations indicate that PRPF19 downregulation causes a switch of the *MDM4* splicing isoform from MDM4-FL to MDM4-S during replicative senescence.

We next investigated whether MDM4 participates in activation of the p53–p21 pathway in PRPF19-depleted cells. MDM4-FL overexpression attenuated activation of the p53–p21 pathway in PRPF19-depleted cells (Fig. 5, *F* and *G*). To further examine the effect of MDM4 on replicative senescence, we serially cultured luciferase-expressing and MDM4-FL-overexpressing TIG-3 cells. Intriguingly, in comparison with luciferase-expressing TIG-3 cells, MDM4-FL-overexpressing TIG-3 cells delayed the entry into senescence state (Fig. 5, *H* and *I*). Taken together, these results indicate that PRPF19 downregulation stimulates activation of the p53–p21 pathway by modulating *MDM4* alternative splicing.

### PRPF19 regulates *MDM4* alternative splicing by modulating the physical interaction between PRPF3 and PRPF8

**Figure 6:**
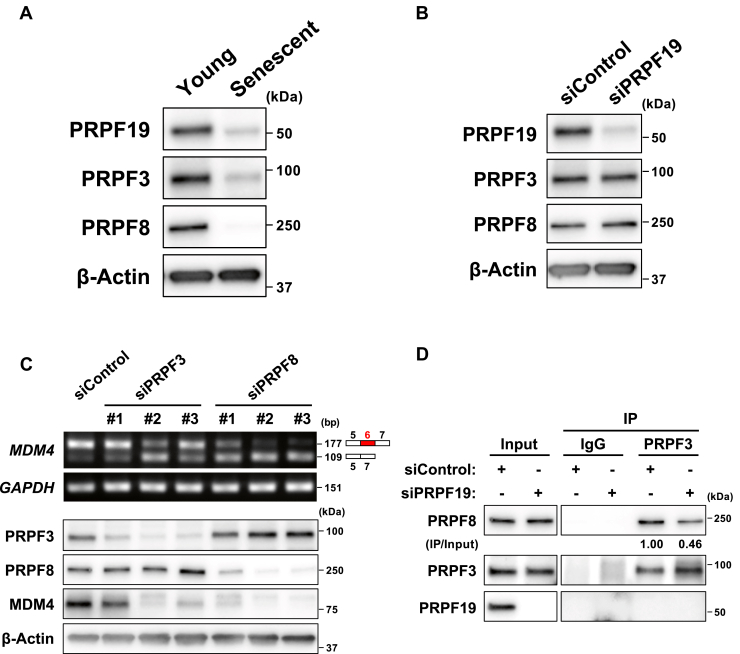
**U4/U6.U5 tri-snRNP-associated proteins are necessary for inclusion of exon 6 in the *MDM4* mRNA**. ***A*,** immunoblot analysis of young and senescent TIG-3 cells. ***B*,** immunoblot analysis of TIG-3 cells 3 days after transfection with the indicated siRNAs. ***C*,** RT-PCR analysis (*upper*) and immunoblot analysis (*bottom*) of TIG-3 cells 3 days after transfection with the indicated siRNAs. ***D*,** co-immunoprecipitation analysis, using anti-PRPF3 antibody, of TIG-3 cells 3 days after transfection with the indicated siRNAs.

Finally, we investigated the molecular mechanism by which PRPF19 regulates *MDM4* alternative splicing during cellular senescence. PRPF3 and PRPF8 are critical components of U4 snRNP and U5 snRNP, respectively (26). PRPF19 promotes the physical interaction of PRPF3 with PRPF8, which leads to stabilization of the U4/U6.U5 tri-snRNP, one of the core building blocks of the spliceosome (36). Therefore, we hypothesized that disassembly of the U4/U6.U5 tri-snRNP causes a switch of the *MDM4* splicing isoform from MDM4-FL to MDM4-S. To test this hypothesis, we first examined the expression of PRPF3 and PRPF8 in senescent or PRPF19-knockdown TIG-3 cells. Importantly, PRPF3 and PRPF8 were downregulated in senescent TIG-3 cells, whereas PRPF19 knockdown had little effect on the expression of PRPF3 and PRPF8 in young TIG-3 cells (Fig. 6, *A* and *B*).

To further investigate the roles of PRPF3 and PRPF8 for MDM4 splicing, we examined the splicing alteration of *MDM4* mRNA after knockdown of PRPF3 or PRPF8. As observed in PRPF19-depleted cells, knockdown of PRPF3 or PRPF8 also caused exon 6 skipping in the *MDM4* mRNA and decreased the level of MDM4 protein (Fig. 6*C*). These observations indicate that the U4/U6.U5 tri-snRNP-associated proteins play important roles in triggering cellular senescence.

Since the ubiquitination of PRPF3 by PRFP19 is required for its interaction with PRPF8 (36), we investigated whether PRPF19 knockdown inhibits the physical interaction between PRPF3 and PRPF8 in TIG-3 cells. To this end, we performed co-immunoprecipitation (co-IP) with anti-PRPF3 antibody in PRPF19-depleted TIG-3 cells. As expected, PRPF19 knockdown weakened the interaction between PRPF3 and PRPF8 (Fig. 6*D*). These observations suggest that PRPF19 regulates *MDM4* alternative splicing by modulating the physical interaction between PRPF3 and PRPF8. Given that PRPF3 and PRPF8 were downregulated in senescent TIG-3 cells, these findings also indicate that PRPF19 plays a functional role in an early step in cellular senescence.

**Figure 7:**
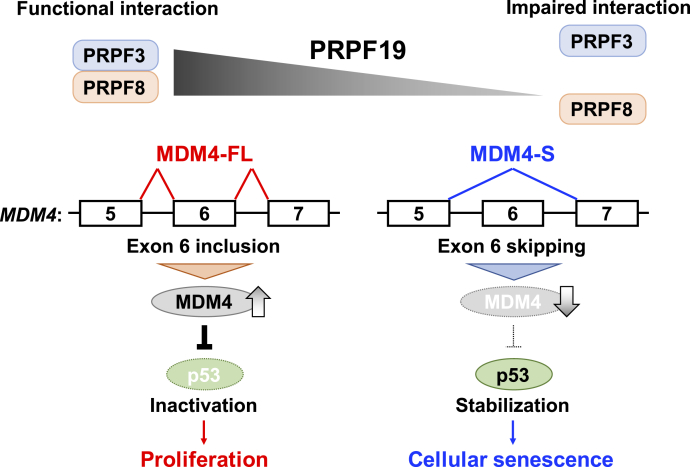
**Model of cellular senescence caused by PRPF19 downregulation in human fibroblasts.** PRPF19 is downregulated during replicative senescence in normal human fibroblasts. PRPF19 downregulation alters the MDM4 splicing isoform from MDM4-FL to MDM4-S by weakening the physical interaction between PRPF3 and PRPF8, which leads to induction of p53–p21-dependent cellular senescence.

Collectively, our results indicated that PRPF19 is a critical regulator for the RNA splicing machinery, which determines the cell fate transition from proliferation to senescence in human fibroblasts (Fig. 7).

## Discussion

Since cellular senescence is associated with various biological phenomena, including age-related disease, embryonic development, and tissue repair, it is thought to play physiological roles in tissue homeostasis and disease (6). Therefore, elucidation of the signaling pathways or molecular mechanisms that induce senescence is important for understanding the functional contribution of this phenomenon in each biological context. In this study, we identified PRPF19 as a key regulator of cellular senescence in normal human fibroblasts. We observed that PRPF19 was significantly downregulated during cellular senescence, concomitant with activation of the p53–p21 pathway. In addition, we found that PRPF19 is involved in the regulation of *MDM4* alternative splicing. PRPF19 knockdown caused exon 6 skipping in the *MDM4* mRNA, resulting in the generation of its unstable isoform. PRPF19 knockdown weakened the interaction between PRPF3 and PRPF8, which are important for the assembly of the U4/U6.U5 tri-snRNP; this, in turn, resulted in exon 6 skipping in *MDM4* mRNA. Thus, our findings indicate that PRPF19 is a critical regulator of p53-dependent cellular senescence.

In senescent cells, multiple changes are observed at the molecular and morphological levels. Several studies reported that dysregulation or alteration of RNA splicing is associated with cellular senescence and aging (25). For instance, p53β, an alternative splicing product of the *TP53* gene, promotes cellular senescence in human fibroblasts (35, 38). More importantly, the splicing factors SRSF3 and SRSF7 are critical for the generation of the p53β isoform in response to DNA damage (24). Consistent with these studies, our microarray and RT-qPCR analyses revealed downregulation of splicing factors, including members of the SR protein family, during replicative senescence. Among them, PRPF19 was downregulated to the greatest extent in senescent TIG-3 cells, and PRFP19 knockdown induced cellular senescence in young TIG-3 cells. Given that PRPF19 knockdown induced senescence-like phenotype independently of the signaling pathways involved in ATM, ATR, and p53β, our results suggest that several distinct pathways in RNA splicing are used selectively in response to the type of stimulation or stress, such as telomere dysfunction, DNA damage, or oxidative stress. To elucidate how these splicing factors are selectively involved in cellular senescence, future studies should investigate the upstream regulators of PRPF19.

MDM4 functions as a negative regulator of p53 and is required for the proper regulation of p53 activity during development (39). In contrast to MDM2, MDM4 is expressed at extremely low levels in adult tissues such as the eye, heart, and lung (40), but at higher levels in various types of cancer cells (13, 14, 15, 16). Because p53 inactivation in cancer cells is frequently a consequence of upregulation of MDM4 at the protein level, reactivation of p53 by MDM4 silencing effectively inhibits tumor cell proliferation (17). Although the underlying mechanisms have not yet been fully elucidated, MDM4 silencing also effectively inhibits the proliferation of cells expressing mutant p53 (41, 42). Together, these observations strongly indicate that MDM4 is a promising target for cancer therapy. However, one of the major challenges of evaluating MDM4 expression is the lack of correlation between its expression at the mRNA and protein levels. The *MDM4* gene produces two main isoforms, MDM-FL and MDM4-S; the latter is unstable. Because the ratio of MDM4-FL to MDM4-S mRNA is correlated with the level of MDM4 protein in several tumor cell lines, this ratio is considered more useful for investigations of MDM4 expression in target tissues or cells. The splicing enhancer SRSF3 is necessary for the generation of MDM4-FL in melanoma cells (12). Although SRSF3 was slightly downregulated in senescent TIG-3 cells, we confirmed that SRSF3 knockdown induced a switch in alternative splicing from MDM4-FL to MDM4-S. Together with our results, these lines of evidence suggest that these splicing factors might be important determinants of the ratio of MDM4-FL to MDM4-S and that their expression is likely to be tightly controlled according to cell type or condition. Given that the endogenous expression of *PRPF19* mRNA was correlated with MDM4 expression at the protein level in human fibroblasts, as well as the ratio of MDM4-FL to MDM4-S, investigation of *PRPF19* mRNA expression could enable more precise evaluation of MDM4 protein expression. Therefore, further investigation of the relationship between PRPF19 and MDM4 in cancer cells would contribute to improvements in cancer diagnosis and therapy.

The primary role of RNA splicing is removal of noncoding introns by large complexes composed of the major and minor spliceosomes (26). The major spliceosome consists of five snRNPs (U1, U2, U4, U5, and U6, which tightly binds to U4). After initial recognition of the splice sites by U1 and U2 snRNPs, the U4/U6.U5 tri-snRNP joins this complex, triggering drastic conformational changes and protein exchanges. These remarkable changes induced by the U4/U6.U5 tri-snRNP enable catalytic core formation on the spliceosome and completion of the splicing reaction. The stability of the U4/U6.U5 tri-snRNP is mainly dependent on the physical interaction between PRPF3 and PRPF8, which are components of the U4 and U5 snRNPs, respectively (36). Consistent with a previous study, we confirmed that PRPF19 stabilized the U4/U6.U5 tri-snRNP by regulating the interaction between PRPF3 and PRPF8. More importantly, although PRPF19 knockdown had little effect on the expression level of PRPF3 and PRPF8 at 72 h after transfection with siRNAs, PRPF3 and PRPF8 were downregulated in the senescent state with PRPF19 downregulation. Previous study reported that senescence-associated protein degradation (SAPD) promotes selective degradation of proteins associated with cell cycle and RNA metabolism in senescent cells (43). Therefore, their findings and our results provide the possibility that PRPF3 and PRPF8 might be a target of SAPD. While further studies are needed to understand the regulation of expression changes of RNA splicing factors across senescence process, our findings indicate that PRPF19-mediated regulation of the U4/U6.U5 tri-snRNP is critical for an early step of cellular senescence.

In conclusion, our study demonstrated that PRPF19 plays a functional role in cellular senescence *via* activating the p53–p21 signaling pathway. Since accumulating evidence suggests that senolytic therapies (*i.e.*, approaches that eliminate senescent cells) improve senescence-associated pathologies and extend healthy life expectancy (44, 45, 46), our findings provide novel insights regarding the RNA splicing machinery underlying cellular senescence and raise the possibility that modulation of RNA splicing might be a promising approach for senolytic therapy.

## Experimental procedures

### Cell culture

TIG-3 (Health Science Research Resources Bank) and Lenti-X 293T cells (Clontech/Takara Bio) were maintained in Dulbecco’s modified Eagle’s medium (Sigma-Aldrich) supplemented with 10% fetal bovine serum (FBS) (GE Healthcare), antibiotic–antimycotic; 50 U/ml penicillin, 50 μg/ml streptomycin, and 125 μg/ml amphotericin B (Gibco). MRC-5 and IMR-90 cells (National Institutes of Biomedical Innovation, Health and Nutrition) were maintained in Minimum Essential Medium Eagle (Sigma-Aldrich) supplemented with 10% FBS, antibiotic–antimycotic, 50 U/ml penicillin, 50 μg/ml streptomycin, and 125 μg/ml amphotericin B. The cells were cultured under 5% CO_2_ at 37 °C. Under our culture conditions, TIG-3, MRC-5, and IMR-90 cells reached replicative senescence at approximately PDL 80, 75, and 58, respectively. TIG-3 cells at PDL 41–59 and 77–83 were used as young and senescent cells, respectively. MRC-5 cells at PDL 50–55 and 70–75 were used as young and senescent cells, respectively. IMR-90 cells at PDL 40–45 and 55–58 were used as young and senescent cells, respectively.

### Plasmid DNA for viral infection

The lentiviral cDNA expression vector pCDH-CMV-EF1-GFP-T2A-Puro (System Biosciences, LCC) was used to establish TIG-3 cells overexpressing PRPF19 or MDM4. PRPF19 and MDM4 expression vectors were constructed by insertion of the coding region of the *PRPF19* and *MDM4* sequences, respectively, at the *Eco*RI and *Not*I sites of pCDH-CMV-EF1-GFP-T2A-Puro, using the In-Fusion HD cloning kit (Takara Bio). For lentivirus production, Lenti-X 293T cells were cotransfected with PRPF19 or MDM4 expression vectors and the ViraPower lentiviral expression system (Invitrogen) using Lipofectamine LTX and PLUS reagent (Invitrogen). Forty-eight hours after transfection, culture medium containing lentivirus was collected and concentrated using a Lenti-X concentrator (Clontech). TIG-3 cells were infected with lentivirus for 24 h. The cells were maintained in complete growth medium containing 1 μg/ml puromycin.

### Microarray analysis

Total RNA was purified using ISOGEN II (NIPPON GENE). Microarray analysis of mRNA levels was performed using 3D-Gene microarrays (TORAY). Pathway and Gene Ontology analyses were performed using GenMAPP ver. 2.1 (MAPP Finder) and GENECODIS2.0, respectively.

### RT-qPCR analysis

Total RNA was purified using the miRNeasy Mini kit (QIAGEN) or ISOGEN II (NIPPON GENE). For analysis of mRNA levels, reverse transcription was performed using the High-Capacity RNA-to-cDNA kit (Applied Biosystems), and real-time PCR was performed using the KAPA SYBR FAST qPCR Master Mix (2×) Universal (KAPA Biosystems) on a Rotor-Gene Q (QIAGEN). Relative expression levels were calculated based on the 2^−ΔΔCt^ method. PCR primers are listed in Supplementary Table 1.

### Immunoblot analysis

Cells were lysed in 2× SDS sample buffer (116.7 mM Tris-HCl [pH 6.8], 3.67% [w/v] SDS, 0.004% [w/v] BPB, 12% [v/v] glycerol, 200 mM DTT). To denature proteins, the cell lysate was boiled at 95 °C for 5–10 min. Proteins were separated by SDS-PAGE and transferred from gels to PVDF membranes. Membranes were incubated at 4 °C overnight with the following primary antibodies: anti-PRPF19 (1:1000; Bethyl Laboratories, A300-101A), anti-CWC22 (1:1000; Santa Cruz Biotechnology, Dallas, TX, USA, sc-398178), anti-WTAP (1:1000; Santa Cruz Biotechnology, sc-374280), anti-SRSF1 (1:1000; Santa Cruz Biotechnology, sc-33652), anti-DHX15 (1:1000; Santa Cruz Biotechnology, sc-271686), anti-p53 (1:1000; Santa Cruz Biotechnology, sc-126), anti-phospho-p53 (1:1000; Cell Signaling Technology, #9284), anti-p21 (1:1000; BD Pharmingen, #554228), anti-p16 (1:1000; Cell Signaling Technology, #92803), anti-cyclin D1 (1:1000; Cell Signaling Technology, #2926), anti-Cyclin D3 (1:1000; Cell Signaling Technology, #2936), anti-CDK4 (1:1000; Cell Signaling Technology, #2906), anti-CDK6 (1:1000; Cell Signaling Technology, #3136), anti-ATR (1:1000; Santa Cruz Biotechnology, sc-1887), anti-ATM (1:1000; Merck Millipore, PC116), anti-SRSF3 (1:1000; Medical & Biological Laboratories, RN080PW), anti-MDM4 (1:1000; Merck Millipore, #04-1555), anti-PRPF3 (1:1000; Proteintech, 10106-1-AP), anti-PRPF8 (1:1000; Santa Cruz Biotechnology, sc-55533), or anti-β-Actin (1:5000; Sigma-Aldrich, A5441). Next, the membranes were incubated at room temperature for 1 h with secondary antibody: horseradish-peroxidase-conjugated IgG, anti-mouse or rabbit (1:5000; Jackson ImmunoResearch Laboratories) or anti-goat (1:5000; Santa Cruz Biotechnology). Chemiluminescence signals were detected using Western Lightning Plus-ECL (PerkinElmer) or ImmunoStar Zeta (FUJIFILM Wako Pure Chemical Co) and imaged on an ImageQuant LAS 4000mini (GE Healthcare) or FUSION SYSTEM (Vilber-Lourmat). The chemiluminescence signal intensity was quantified using the ImageJ software.

### siRNA transfection

Cells were reverse-transfected with siRNA using Lipofectamine RNAiMAX Transfection Reagent (Invitrogen). The final concentration of siRNA was 10 nM. The siRNA lists are indicated in Supplementary Table 2.

### SA-β-gal activity assay

SA-β-gal activity was assessed using the fluorescence-based SPiDER-βGal kit (Dojindo). After cells were precultured in growth medium supplemented with 100 nM bafilomycin A1 for 1 h, 1 μM SPiDER-βGal was added, and the cells were cultured for 15 min. Subsequently, the cells were washed twice with PBS(-) and harvested by trypsinization. Flow cytometry was performed on a FACSCalibur (BD Biosciences) to detect fluorescence-based β-gal activity. For each sample, 10,000 single-cell events were collected by gating on forward scatter and side scatter, and an FL1 histogram was generated. Finally, data were analyzed using FlowJo (FlowJo LLC).

### Detection of SAHFs

We detected senescence-associated heterochromatin foci (SAHFs) as described previously with minor modifications (47). Cells prepared in chamber slides were fixed in 4% paraformaldehyde for 10 min and then permeabilized with 0.2% Triton-X 100 for 5 min. After blocking cells in 3% BSA for 5 min, the cells were immunostained with anti-trimethyl-histone H3 (Lys9) (1:500; Merck Millipore, 07-422) as the primary antibody for 2 h, followed by Alexa Fluor 594-conjugated anti-rabbit IgG (1:1000; Invitrogen) as the secondary antibody for 1 h. To stain DNA, cells were incubated in 0.15 μg/ml DAPI for 3 min. After washing, cells were soaked in fluorescence mounting medium (Dako) and covered with a coverslip. SAHFs were observed on a BZ-X810 All-in-One Fluorescence Microscope (Keyence).

### EdU cell proliferation assay

For EdU cell proliferation assays, cells transfected with siRNA were prepared in 24-well plates. Cells incorporated EdU for 24 h and were then stained using the Click-iT Plus EdU Alexa Fluor 488 Imaging kit (Molecular Probes). Cells were observed on a BZ-X810 All-in-One Fluorescence Microscope.

### Detection of single-stranded DNA

Single-stranded DNA was detected as described previously with minor modifications (30). Cells were precultured in growth medium supplemented with 20 μM BrdU for 48 h. After siRNA transfection, cells were fixed in 70% ethanol. To detect exposed DNA strands, cells were immunostained with anti-BrdU (1:200; Dako) as the primary antibody at 37 °C for 1 h, followed by Alexa Fluor 488-conjugated anti-mouse IgG (1:1000; Invitrogen) at room temperature for 1 h. For flow cytometry, after two washes in PBS(-), cells were stained with 50 μg/ml propidium iodide for 30 min at RT in the presence of 100 μg/ml RNase A. Data were acquired and analyzed on a FACSCalibur.

### Comet assay

Cells were trypsinized and subjected to an alkaline comet assay using the Comet Assay kit (Trevigen). The percentage of DNA in the tail was measured using TriTek Comet Score (ver. 1.5) software (TriTek Corp.)

### Splicing isoform analysis by RT-PCR

Total RNA was purified using the miRNeasy Mini kit, and reverse transcription was performed using the High-Capacity RNA-to-cDNA kit. Alternative splicing of *TP53* and *MDM4* was analyzed using KOD -plus- ver. 2 (TOYOBO). The three *TP53* isoforms (α, β, and γ) were visualized using a forward primer that bound in exon 7 and a reverse primer that bound in exon 10. The two *MDM4* isoforms (MDM4-FL and MDM4-S) were visualized using a forward primer that bound in exon 5 and a reverse primer that bound in exon 7. PCR conditions were as follows: predenature, 94 °C for 2 min; 30 cycles of denaturation (98 °C for 10 s), annealing (57 °C for 30 s), and extension (68 °C for 30 s). PCR primers are listed in Supplementary Table 1.

### RNA sequence analysis

Total RNA was purified using the miRNeasy Mini kit (QIAGEN) with RNase-free DNase (QIAGEN). Library preparation, sequencing, and data analysis were performed by DNAFORM (Yokohama). Quality and quantity of extracted RNA were assessed on a NanoDrop 8000 Microvolume UV-Vis spectrophotometer (Thermo Fisher Scientific) and a BioAnalyzer 2100 System using the Agilent RNA 6000 Nano kit (Agilent Technologies). RNA-seq libraries were prepared using the SMARTer Stranded Total RNA Sample Prep Kit - HI Mammalian (Takara Bio). In brief, ribosomal RNA was depleted using RiboGone, which was included in the kit. Next, first-strand cDNA synthesis was performed using the N6 primer. First-strand cDNA was purified using an equal volume of AMPure XP beads (Beckman Coulter). The purified cDNA was PCR-amplified into Illumina-specific RNA-seq libraries, which were sequenced on a HiSeq sequencer (Illumina) to generate 150 nt paired-end reads. The quality of sequence data was first assessed using FastQC (ver. 0.11.7). Raw sequence reads were trimmed and quality-filtered with the Trim Galore! (ver. 0.4.4), Trimmomatic (ver. 0.36), and cutadapt (ver. 1.16) software. Trimmed reads were mapped to the human reference genome (GRCh38.p10) using STAR (ver. 2.6.1a). Differential alternative splicing events were detected using rMATS (ver. 4.0.2). Genome annotation showing the *MDM4* splice form was acquired using the Integrative Genomics Viewer (ver. 2.4.16).

### Co-immunoprecipitation

Cells were lysed in 1× cell lysis buffer (25 mM Tris-HCl [pH 7.5], 150 mM NaCl, 1% [v/v] NP-40, 1 mM EDTA, 5% [v/v] glycerol, protease inhibitors). The cell lysates were precleared with Protein G beads (GE Healthcare) at 4 °C for 1 h. The precleared cell lysates were incubated with 2 μg normal rabbit IgG (Santa Cruz Biotechnology, sc-2027) or anti-PRPF3 at 4 °C overnight and then with Protein G beads at 4 °C for 2 h. The beads were washed three times with cell lysis buffer, and proteins were eluted from the beads with SDS sample buffer. The eluents were carried to immunoblot analysis.

### Statistical analysis

Data were analyzed using the GraphPad Prism 8 software (GraphPad Software). All data in bar graphs are presented as means ± SD of three independent experiments. Differences between two groups were statistically analyzed using the paired or unpaired Student’s *t* test. *p*-values are indicated as follows: not significant (n.s.), ≥0.05; ∗< 0.05; ∗∗< 0.01; and ∗∗∗< 0.001.

## Data availability

The gene expression profiles in replicative senescence in TIG-3 cells and RNA sequencing profiles of control and PRPF19-knockdown TIG-3 cells have been deposited at the Gene Expression Omnibus (GEO) with accession number GSE162201 and GSE168391, respectively.

## Supporting information

This article contains supporting information.

## Conflict of interest

The authors declare that they have no conflicts of interest with the contents of this article.
