# Supplementary material for: PRPF19 regulates p53-dependent cellular senescence by modulating alternative splicing of MDM4 mRNA
Source: J Biol Chem. 2021 Jun 16;297(1):100882. doi: 10.1016/j.jbc.2021.100882 (PMC8274299; doi:10.1016/j.jbc.2021.100882)
Supplement: Figures S1–S4 and Tables S1 and S2 [file mmc1.pdf]

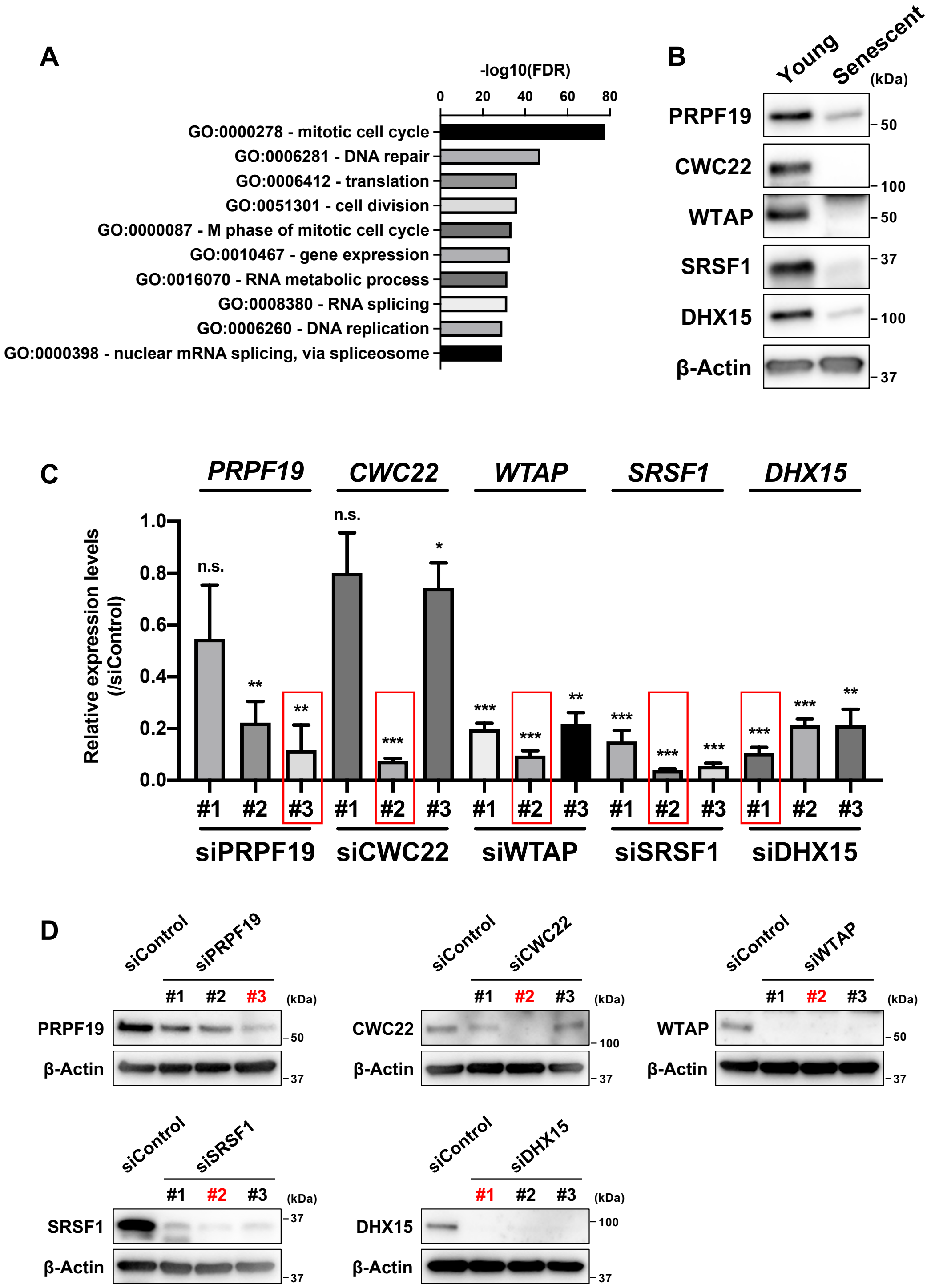

Supplemental Figure 1

A. Top 10 significant Gene Ontology terms containing genes whose expression were down-regulated in senescent cells. **B.** Immunoblot analysis of young and senescent TIG-3 cells. **C.** Relative quantification by RT-qPCR analysis of TIG-3 cells 3 days after transfection with the indicated siRNAs. Expression levels of each gene were normalized against the corresponding level of *GAPDH*. Data represent the means ± SD of three independent experiments. *p < 0.05, **p < 0.01, ***p < 0.001, n.s. (not significant); two-tailed Student's t-test. **D.** Immunoblot analysis of TIG-3 cells 3 days after transfection with the indicated siRNAs.

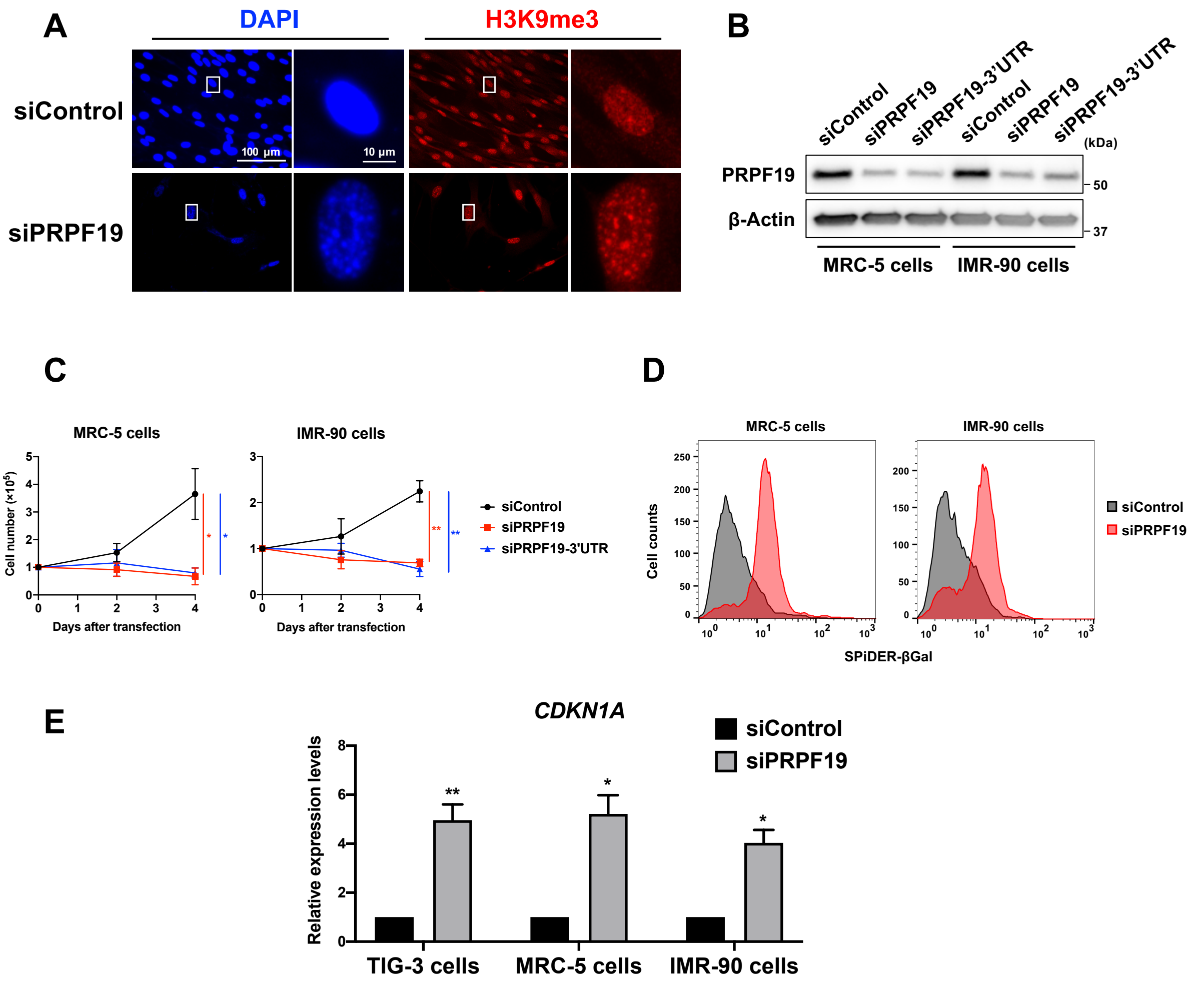

Supplemental Figure 2

A. Fluorescence microphotographs of DAPI and H3K9me3 staining in TIG-3 cells 7 days after transfection. Expanded images show a nucleus in white box. **B.** Immunoblot analysis of MRC-5 and IMR-90 cells 3 days after transfection with the indicated siRNAs. **C.** Cell growth curve in MRC-5 and IMR-90 cells. Cells were counted at the indicated time points after transfection with the indicated siRNAs. All values represent means ± SD of three independent experiments. *$p < 0.05$, **$p < 0.01$; two-tailed Student's t-test. **D.** Histogram shows fluorescence-based SA-β-gal activity of MRC-5 and IMR-90 cells 7 days after transfection with the indicated siRNAs. **E.** Relative quantification by RT-qPCR analysis of TIG-3, MRC-5, and IMR-90 cells 3 days after transfection with the indicated siRNAs. Expression levels of *CDKN1A* were normalized against the corresponding level of *GAPDH*. Data represent the means ± SD of three independent experiments. *$p < 0.05$, **$p < 0.01$; two-tailed Student's t-test.

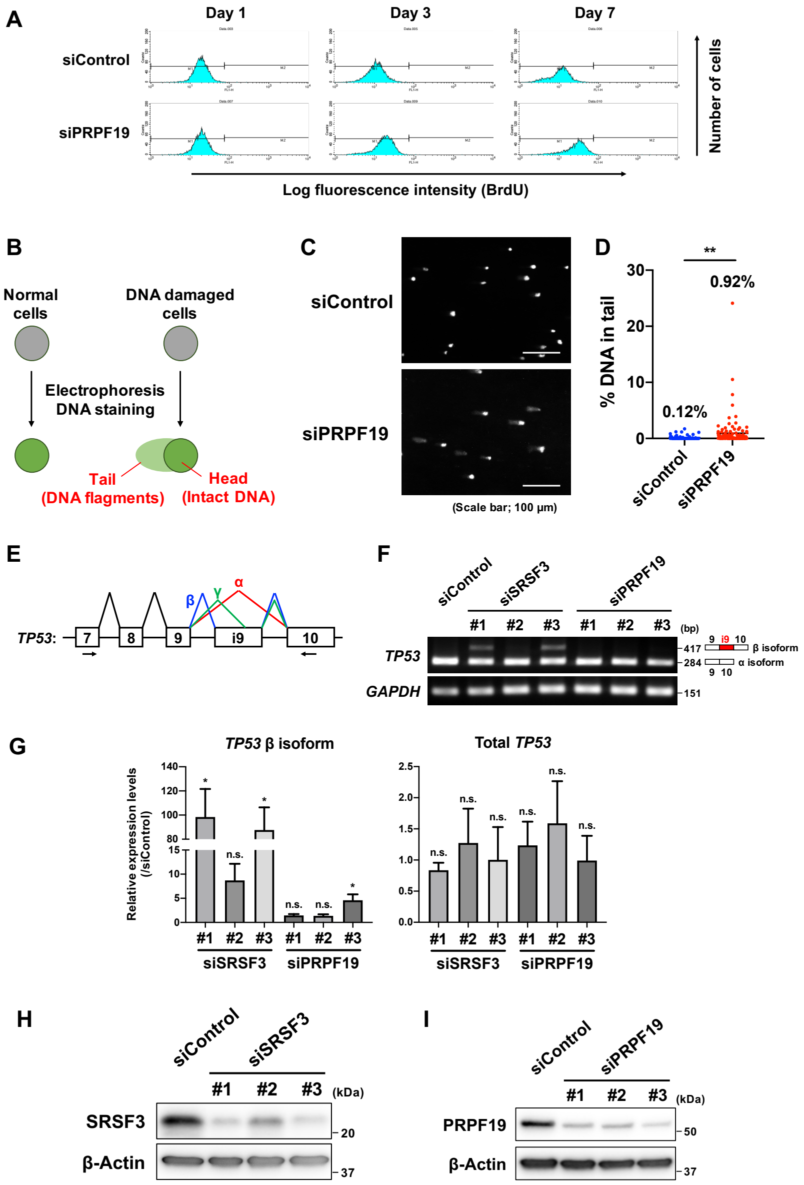

Supplemental Figure 3

A. Histogram shows BrdU fluorescence intensity, based on exposed DNA strand. TIG-3 cells were pre-tcultured in growth medium supplemented with 20 μM BrdU for 48 h prior to transfection with the indicated siRNAs. Cells were immunostained with anti-BrdU under native conditions at the indicated time points after transfection. **B.** Schematic model of comet assay to detect DNA damage by single cell gel electrophoresis. After cells are spotted and lysed on the agarose slide, the damaged DNA fragments are separated from the intact DNA (head) by electrophoresis and form comet tail-like pattern. **C.** Comet assay shows the levels of DNA damage in TIG-3 cells 2 days after transfection with the indicated siRNAs. Scale bar; 100 μm. **D.** Dot plot shows percentage of DNA in the tail in the experiment shown in supplemental figure 4C. **$p < 0.01$; two-tailed Welch's t-test. **E.** PCR primers designed to detect each of the three *TP53* splicing isoforms (α, β, and γ). **F.** RT-PCR analysis of TIG-3 3 days after transfection with the indicated siRNAs. **G.** Relative quantification by RT-qPCR analysis of TIG-3 cells 3 days after transfection with the indicated siRNAs. Expression levels of each gene were normalized against the corresponding level of *GAPDH*. Data represent the means ± SD of three independent experiments. *$p < 0.05$, n.s. (not significant); two-tailed Student's t-test. **H and I.** Immunoblot analysis of TIG-3 cells 3 days after transfection with the indicated siRNAs.

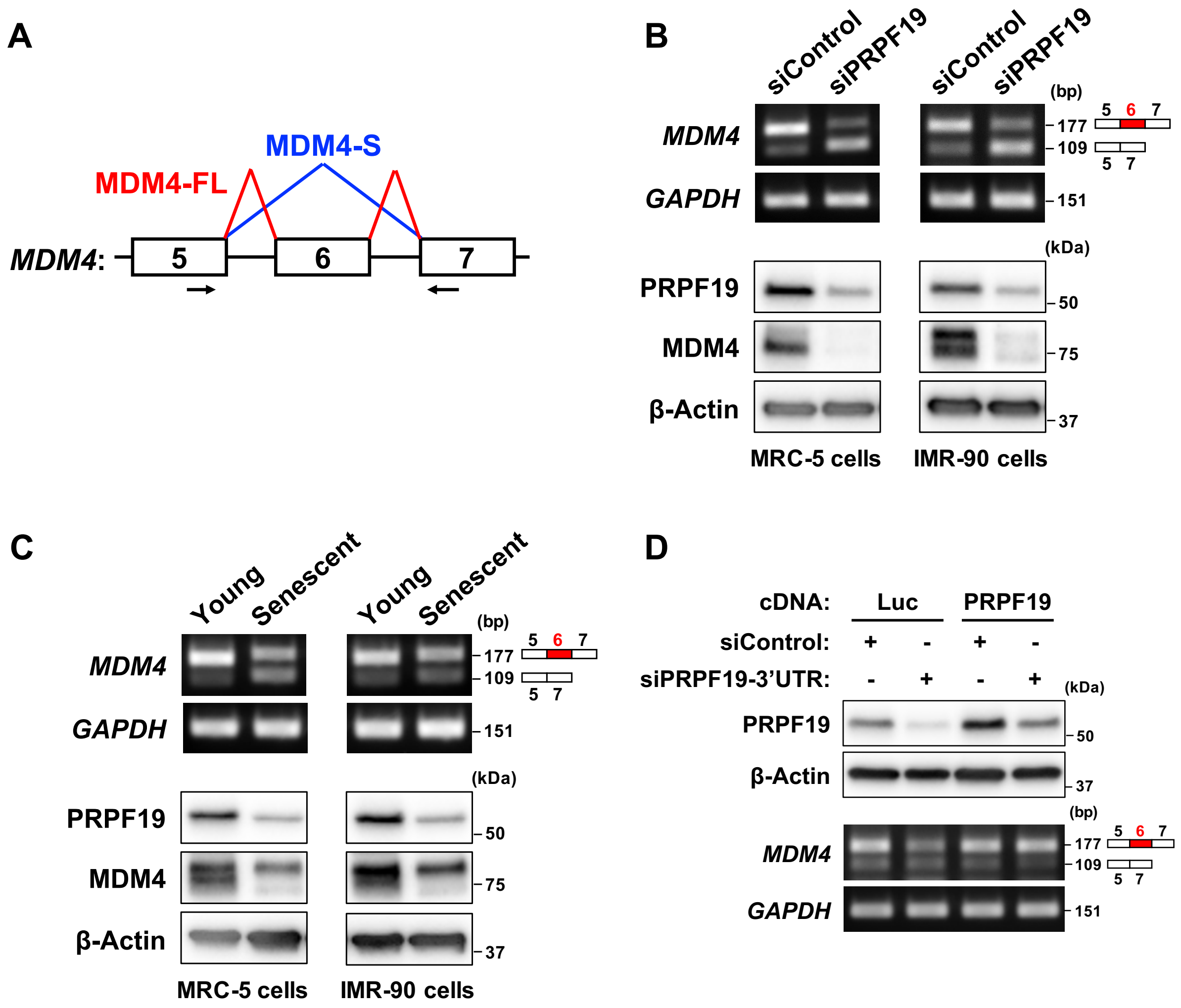

Supplemental Figure 4

A. PCR primers designed to detect each of the two *MDM4* splicing isoforms (MDM4-FL and MDM4-S). **B.** RT-PCR analysis (top) and immunoblot analysis (bottom) of MRC-5 and IMR-90 cells 3 days after transfection with the indicated siRNAs. **C.** RT-PCR analysis (top) and immunoblot analysis (bottom) of young and senescent MRC-5 cells, and young and senescent IMR-90 cells. **D.** Immunoblot analysis (top) and RT-PCR analysis (bottom) of luciferase- or PRPF19-expressing TIG-3 cells 3 days after transfection with the indicated siRNAs.

Table S1. PCR primer lists

Gene	Forward (5'-3')	Reverse (5'-3')
**RT-qPCR**		
GAPDH	CACTTTGTCAAGCTCATTTCCTGG	CTCTTCCTCTTGTGCTCTTGCTGG
SRSF3	CAGTTCATTGTGGGTTGCTTCTG	AGTCTAACAGGGTGGGAGTGTC
PRPF19	ATGGTTACTACCTGGCTACAGCGG	TGCCTGTTGAAGCGATGAACTTGG
PRPF38A	ACCATAGGCAACACCAACACAG	TGGCTTCTTTCACTCACTCTCTCC
CWC22	AGCATACCAGAGGATGAGTTGGG	TGGGTGAAGATTGGAGAAGCAC
WTAP	TCAAGCAAGTCCAGCAGCC	TGTCTTTAGTCTGTTCCAGTTCACC
PRPF4B	TTCTTCTGGTGGGTTTTGCTCC	AGAGACGGGGTGTAGGTTCAG
SRSF1	ACCTCCAGACATCCGAACCAAG	CGAACTCAACGAAGGCGAAGG
DHX15	GCCTGTAAGAGAATAAAGCGTGAAG	GCTGCTGAGGTGGAAGTGTAG
SRSF6	GAAGATAAGCCACGCACAAGCC	ACGGGAGCGACTTTTTGAGATAC
SF3B1	AAAGGCTGCTGGTCTGGCTAC	TGTTGTGTTACGGACATACTCATCC
CDKN1A	TGCAATTCCCCTCTGCTGCTG	TGTGTCCCTTCCCCTTCCAGT
CDKN2A	CAACGCACCGAATAGTTACGGTCG	ACCAGCGTGTCCAGGAAGCCCTC
LMNB1	CTGGAAATGTTTGCATCGAAGA	GCCTCCCATTGGTTGATCC
Total *TP53*	ACTTCTTGTTCCCCACTGACAGC	CCACAACAAAACACCAGTGCAGG
TP53 β isoform	AAATGGTTCTATGACTTTGCCTGATA	CAGCTCTCGGAACATCTCGAA
**RT-PCR**		
GAPDH exon 3-exon 4	TGGTCACCAGGGCTGCTT	AGCTTCCCGTTCTCAGCCTT
TP53 exon 7-exon 10	CTCACCATCATCACACTGGAA	TCATTCAGCTCTCGGAACATC
MDM4 exon 5-exon 7	GAATCTTGTCACTTTAGCCACTGC	TGCTCTGAGGTAGGCAGTGTG

Table S2. siRNA lists

siRNA	Resource
Silencer Select Negative Control No.1 siRNA	Thermo Fisher Scientific, 4390844
Silencer Select siRNA targeting PRPF19 #1	Thermo Fisher Scientific, ID: s223754
Silencer Select siRNA targeting PRPF19 #2	Thermo Fisher Scientific, ID: s26186
Silencer Select siRNA targeting PRPF19 #3	Thermo Fisher Scientific, ID: s26184
Silencer Select siRNA targeting CWC22 #1	Thermo Fisher Scientific, ID: s33633
Silencer Select siRNA targeting CWC22 #2	Thermo Fisher Scientific, ID: s33631
Silencer Select siRNA targeting CWC22 #3	Thermo Fisher Scientific, ID: s33632
Silencer Select siRNA targeting WTAP #1	Thermo Fisher Scientific, ID: s18432
Silencer Select siRNA targeting WTAP #2	Thermo Fisher Scientific, ID: s18431
Silencer Select siRNA targeting WTAP #3	Thermo Fisher Scientific, ID: s18433
Silencer Select siRNA targeting SRSF1 #1	Thermo Fisher Scientific, ID: s12727
Silencer Select siRNA targeting SRSF1 #2	Thermo Fisher Scientific, ID: s12726
Silencer Select siRNA targeting SRSF1 #3	Thermo Fisher Scientific, ID: s12725
Silencer Select siRNA targeting DHX15 #1	Thermo Fisher Scientific, ID: s4030
Silencer Select siRNA targeting DHX15 #2	Thermo Fisher Scientific, ID: s4029
Silencer Select siRNA targeting DHX15 #3	Thermo Fisher Scientific, ID: s4028
Silencer Select siRNA targeting SRSF3 #1	Thermo Fisher Scientific, ID: s12733
Silencer Select siRNA targeting SRSF3 #2	Thermo Fisher Scientific, ID: s12731
Silencer Select siRNA targeting SRSF3 #3	Thermo Fisher Scientific, ID: s12732
Silencer Select siRNA targeting PRPF3 #1	Thermo Fisher Scientific, ID: s17434
Silencer Select siRNA targeting PRPF3 #2	Thermo Fisher Scientific, ID: s17435
Silencer Select siRNA targeting PRPF3 #3	Thermo Fisher Scientific, ID: s17436
Silencer Select siRNA targeting PRPF8 #1	Thermo Fisher Scientific, ID: s20797
Silencer Select siRNA targeting PRPF8 #2	Thermo Fisher Scientific, ID: s20798
Silencer Select siRNA targeting PRPF8 #3	Thermo Fisher Scientific, ID: s20796
Stealth RNA siRNA Negative Control Med GC Duplex #2	Thermo Fisher Scientific, 12935-112
Stealth siRNA targeting PRPF19	Thermo Fisher Scientific, ID: HSS120653
Stealth siRNA targeting PRPF19-3'UTR	Thermo Fisher Scientific,<br>sense: 5'- UGUAAGCAGUGAUCUAGUUUCAUUA-3',<br>antisense: 5'- UAAUGAAACUAGAUCACUGCUUACA-3'
Stealth siRNA targeting TP53	Thermo Fisher Scientific,<br>sense: 5'- CCAGUGGUAAUCUACUGGGACGGAA-3',<br>antisense: 5'- UUCCGUCCCAGUAGAUUACCACUGG-3'
Stealth siRNA targeting MDM4	Thermo Fisher Scientific, ID: HSS106419
AllStars Negative Control siRNA	QIAGEN, 1027280
siRNA targeting ATR	Hokkaido System Science,<br>sense: 5'- CCUCCGUGAUGUUGCUUGAtt-3',<br>antisense: 5'- UCAAGCAACAUCACGGAGGtt-3'
siRNA targeting ATM	Hokkaido System Science,<br>sense: 5'- GCGCCUGAUUCGAGAUCCUtt-3',<br>antisense: 5'- AGGAUCUCGAAUCAGGCGCtt-3'
